# Protocol-driven approach of bleeding abdominal and pelvic trauma

**DOI:** 10.1186/1749-7922-1-17

**Published:** 2006-06-17

**Authors:** Osvaldo Chiara, Stefania Cimbanassi, Fabio Castelli, Rosario Spagnolo, Paolo Girotti, Giacinto Pizzilli, Alessio Pitidis, Sara Andreani, Raffaele Pugliese, Dario Capitani

**Affiliations:** 1 Dipartimento di Emergenza Accettazione – Trauma Team Ospedale Niguarda Ca'Granda Milano, Scuola di Specializzazione in Chirurgia Generale, Universita' degli Studi di Milano, Italy; 2 Divisione di Orto-Traumatologia, Ospedale Niguarda Ca'Granda Milano, Scuola di Specializzazione in Ortopedia, Universita' degli Studi di Milano, Italy; 3 Istituto Superiore di Sanita' del Ministero della Salute, Roma, Italy; 4 Divisione di Chirurgia Generale e Videolaparoscopica Scuola di Specializzazione in Chirurgia Generale, Universita' degli Studi di Milano, Italy

## Background

The considerable force required to disrupt pelvic bones is often associated with abdominal injuries which may be sources of important bleeding themselves. A complex challenge to trauma surgeon is the choice of clinical pathway in management of hemodynamic unstable patients with pelvic ring disruption and potential intraperitoneal or extra-pelvic hemorrhage. Clinical evaluation allows the detection of external hemorrhage and antero-posterior chest x-ray and tube thoracostomy are sufficient to rule out significant hemothorax [1]. Emergency room abdominal ultrasound (US) or diagnostic peritoneal lavage (DPL) may be inconclusive in quantitation of intraperitoneal free fluid and false positive studies may result from retroperitoneal hematoma that leaks blood into peritoneal cavity [2-4]. Contrast enhanced spiral computed tomography (CESCT) offers a complete imaging assessment of the abdomen and pelvis with the best sensitivity and specificity, including injuries of intra- and retroperitoneal organs, soft tissues and bones [5], but may be harmful in unstable hemodynamic conditions. While abdominal bleeding injuries need to be managed with an emergency laparotomy [6], pelvic hemorrhage is optimally treated by angiography/embolization in case of arterial bleeding [7] or pelvic volume closure when venous blood loss from cancellous bony fragments is the main source of bleeding [8,9]. CESCT is very usefull to differentiate pelvic venous or arterial bleeding and may also help to localize bleeding arterial branch with the "contrast extravasation sign" [5,10-12].

In the present study, abdominal and pelvic trauma patients with unstable hemodynamics admitted during a 16 months period have been reviewed. Patients have been prospectively managed with a protocol-driven approach based on pelvic fracture pattern and emergency US. Our hypotheses were the followings: *(a) *in multi-trauma bleeding patients with pelvic fractures causing mechanical instability, a main source of hemorrhage is usually the pelvic fracture itself, while in patients with mechanical stable pelvic fractures, the priority is to search and to treat extrapelvic sources of hemorrhage; *(b) *CESCT is crucial in the selection of appropriate emergency treatment in the case of mechanically unstable bleeding pelvic injury.

## Materials and methods

### Location and study population

The Emergency Department of the Niguarda Ca'Granda Hospital in Milan, Italy, is the referral Trauma Center for an urban area of more than one million inhabitants, evaluating approximately 3,000 injured patients per year. Patients admitted as major trauma, according to American College of Surgeons pre-hospital triage criteria [13], are immediately evaluated by a multidisciplinary team in a dedicated room where ABC resuscitation, plain radiographs, abdominal US/DPL may be all performed. A four-detector spiral CT-scan (Siemens Somatom) and an angiographic suite are both available in rooms close to admitting area, equipped as mini-intensive care units with monitoring and ventilating devices. Protocol of CESCT utilizes 2.5 mm slices and 150 ml of contrast with 3.5 ml/sec injection rate and two scans with a delay of 30 (arterial phase) and 75 (venous phase) seconds.

All major trauma patients admitted in the period between October 1^st^, 2003 and January 31^st^, 2005 (16 months) with abdominal-pelvic trauma and unstable hemodynamics were reviewed for this study. Patients were selected *(i) *if affected by pelvic bone fracture and concomitant serious (coded 3 or more at abbreviated injury score)[14] abdominal injury, *(ii) *if systolic blood pressure at admission was less than 90 mmHg or maintained higher than 90 mmHg with infusions/transfusions or vasopressor support. Standard trauma registry data, demographics, Injury Severity Score (ISS), admission Revised Trauma Score (RTS), TRISS calculated probability of survival (Ps)[15], imaging studies, surgical procedures, outcomes and causes of deaths have been all recorded. Death was attributed to central nervous system (CNS) trauma when lethal brain, brain stem or high C-spine injury were present.

### Clinical protocol

Patients with abdominal-pelvic trauma were initially evaluated by a team including general surgeon, anesthesiologist, orthopedic surgeon, radiologist and nurses, all in house available 24 hours a day. Interventional radiologists provided continuous coverage with immediate availability during working hours and one hour response time off-working hours. All patients were routinely connected to non-invasive blood pressure monitoring, transcutaneous oxygen monitor and end tidal CO2 detector. Hemoglobin levels and arterial blood gases were frequently checked. Arterial and central venous lines were inserted as soon as possible. After initial resuscitation according standard Advanced Trauma Life Support protocol, antero-posterior chest and pelvis radiograph, and abdominal US were obtained. All external bleeding sources were controlled, displaced fractures aligned and chest tube positioned if needed. Orthophedic surgeon classified on plain radiograph pelvic fracture using a simplified Young & Burgess classification. Fracture patterns have been differentiated in two groups: *(i) *antero-posterior compression (APC) fractures type I and lateral compression (LC) fractures type I were considered stable pelvic fractures (SPF), *(ii) *APC fractures type II and III, LC fractures type II and III and all vertical shear (VS) fractures were classified unstable pelvic fractures (UPF). In APC II and III fractures a wide belt around the pelvis (pelvic binder) and taping of knees were positioned. In VS fractures pelvic closure with binder was associated with traction of cranially displaced leg. Abdominal US or supraumbelical DPL (if US unreliable), established the need for celiotomy:

a. If US showed more than 1 cm of fluid strip or in two or more spaces or expanding in serial examinations[6], or DPL obtained more than 10 ml of blood immediately evacuated)[1], celiotomy was indicated, while maintaining pelvic binder, and abdominal injuries were treated. External fixation (EF) was positioned at the end of surgery when indicated by fracture morphology. In case of persistently unstable hemodynamic, angiography was done after surgery with angiographic embolization (AE) as needed.

b. If US was negative or minimally positive, efforts have been made to maintain blood pressure (pelvic binder, infusions/transfusions) and CESCT was performed to detect the presence of venous bleeding or arterial contrast extravasation in the context of pelvic fracture. In case of arterial extravasation from pelvic tissues, AE was used to control pelvic bleeding. The venous bleeding from bone fractures was treated by mechanical pelvic stabilization/compression with EF. When CESCT demonstrated arterial hemorrhage from abdominal parenchyma, AE was used to control bleeding vessels.

Pelvic packing was indicated as life-saving procedure in severe pelvic hemorrhage non-responding to pelvic closure procedures, independently from the need of celiotomy. Recombinant-activated factor seven (rFVIIa, NovoSeven^®^, Novo Nordisk A/S, Bagsvaerd, Denmark) was indicated as pro-coagulant drug in persistent bleeding from pelvic or abdominal injuries, after failure of surgical, angiographic and medical standard treatments.

### Data analysis

Patients with UPF or SPF were compared. Pelvic hemorrhage was considered significant when CESCT and angiography showed active bleeding from pelvis, requiring treatment with AE, EF or other pelvic closure procedures. Celiotomy or CESCT were used to diagnose abdominal sources of bleeding which were considered significant if requiring surgery or AE. In patients arrived in extremis and deceased in emergency room before procedures, balance of injuries was obtained by autopsy. Continuous data have been expressed as mean values ± standard deviation and were compared using t test of Student. Categorical data have been expressed as numbers and percentages and compared using Fisher's exact test. Sensitivity, specificity, positive (PPV) and negative (NPV) predictive values of fracture patterns as predictors of pelvic fracture bleeding have been calculated. Patients with UPF and retroperitoneal bleeding from pelvis requiring treatment were considered true positive (TP). Patients with SPF without retroperitoneal bleeding were regarded as true negative (TN).

## Results

**Figure 1:**
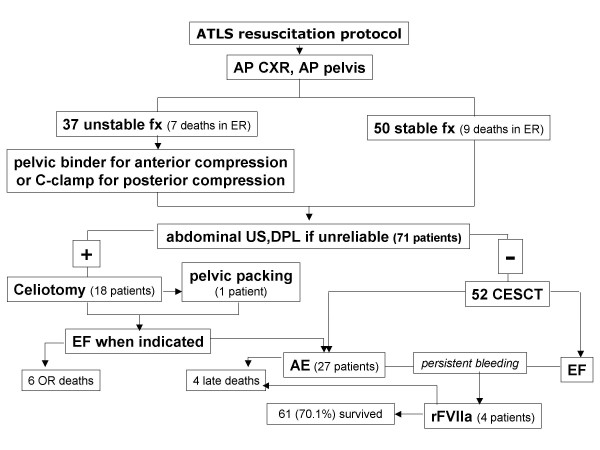
Clinical pathway and outcome of the patient series.

Between October 2003 and January 2005, 87 patients with abdominal-pelvic trauma meeting the study criteria have been selected from trauma registry. Thirthy-seven patients (42.5%) had an unstable and 50 a stable pelvic fracture. Unstable fractures were classified APC II or III in 12 cases, LC II or III in 16, VS in 6 and a combination of previous patterns in 3. Fig. 1 depicts clinical pathway and outcome of this patients series. Sixteen patients were admitted in extremis and died in emergency room before any procedure, six deceased in the perioperative period after celiotomy and 4 during ICU stay. Recombinant FVIIa was used in four cases (three survived) of severe coagulopathy after the failure of surgery, EF, or AE. Demographics were similar in the two groups (table 1). A significant pelvic hemorrhage was observed in 32 of 37 (87%) of UPF and only in 9 of 50 (18%) of SPF patients. Significant abdominal hemorrhage causing hemodynamic instability was discovered in 62% of SPF group and in 16.2% of UPF group. Thereafter, in patients with UPF active intraperitoneal hemorrhage was found significantly less often. Overall mortality of the study population was 29% with 6 of 26 deaths attributable to CNS injuries. Mortality was significantly higher in UPF group while CNS deaths were comparable in the two groups. Associated torso injuries were similar in two groups (table 2). Only urinary tract injuries due to direct pelvic trauma were more often observed in the UPF group.

**Table 1:** Demographics and sites of significant bleeding in unstable and stable pelvic fracture groups.

	***UPF(%)***	***SPF(%)***	***Total (%)***	***p***
*n (%)*	37 (42.5)	50 (57.5)	87 (100)	
*Age*	42.14 ± 16.46	40.78 ± 18.06	41.35 ± 17.32	ns
*ISS*	40.19 ± 13.57	34.65 ± 15.28	36.5 ± 15.7	ns
*RTS*	5.44 ± 2.41	5.94 ± 2.17	5.74 ± 2.27	ns
*Ps*	0.55 ± 0.39	0.62 ± 0.40	0.59 ± 0.39	ns
*Patients with significant pelvic Hemorrhage (%)*	32 (87)	9 (18)	41 (47.12)	<0.01
*Patients with significant abdominal Hemorrhage (%)*	6 (16.2)	31 (62)	37 (42.52)	<0.01
*Total deaths (%)*	18 (48.64)	8 (16)	26 (29)	<0.01
*CNS death (%)*	2 (5.4)	4 (8)	6 (6.8)	ns

**Table 2:** Associated torso injuries in unstable and stable pelvic fracture groups

	***UPF***	***SPF***	***Total (%)***	***p***
*Spleen*	5	7	12 (13.8)	ns
*Liver*	6	5	11 (12.6)	ns
*Kidney*	3	3	6 (6.8)	ns
*Bowel*	2	3	5 (5.7)	ns
*Chest*	21	23	44 (50.57)	ns
*Urinary*	11	1	12 (13.8)	<0.01

If we exclude the emergency room deaths, retroperitoneal bleeding from pelvic fracture was demonstrated in 83.3% of UPF group and 19.51% of SPF group (tab.3). On the basis of CESCT results, in UPF patients emergency AE was necessary for pelvic arterial bleeding in only 9 of 30 (29.8%) of patients, while EF was positioned in emergency to control venous blood loss from the bone in 14 cases. Only in 3 SPF patients AE or EF was considered mandatory to control retroperitoneal blood loss. Therefore, patients requiring emergency AE or EF for retroperitoneal hemorrhage were significantly more common in UPF than in SPF group. A grossly positive US or DPL indicated immediate celiotomy in 18 cases, 4 UPF and 14 SPF patients (p <.01). Emergency celiotomy was therapeutic in all 18 patients, but in three patients intraperitoneal bleeding was spontaneously stopped at the time of surgical exploration. CESCT demonstrated in sixteen patients, mostly in SPF group, bleeding injuries of liver, spleen and kidney amenable to AE. Delayed abdominal surgery was performed in five patients, in two cases for small bowel injuries and in three cases for failures of non-operative management of splenic lacerations. Ten of 71 patients (14%) died: in UPF group four patients deceased for exanguination from multiple injuries of pelvis and abdomen, two for CNS injuries. In SPF group there were two early deaths during laparotomy due to massive bleeding from chest, liver and renal injuries and two late deaths for CNS injuries and multiple organ failure.

Pelvic fracture pattern derived from initial screening radiograph correctly predicted in UPF patients a significant retroperitoneal hemorrhage in 32 of 37 cases, while in SPF patients correctly indicated a significant extra-pelvic source of bleeding in 41 of 50. Therefore, sensitivity was found to be 78.04%, specificity 89.13%, PPV 86.48%, NPV 82%, and overall accuracy 83.9%.

## Discussion

Primary objective of this work was to assess the risk of pelvic hemorrhage in patients with abdominal trauma and pelvic ring fracture. A network of blood vessels lies on the inner wall of the pelvis and can be injured during pelvic trauma. Life threatening hemorrhage is the result of high energy forces with disruption of both bony and ligamentous structures, as occurs in the UPF group. Therefore, a severe hemorrhage due to pelvic blood loss is often associated with pelvic mechanical instability. The Young & Burgess classification of pelvic fractures [12,16] is based on the mechanism of injury with the aim to alert the trauma surgeon of potential sources of bleeding. The more comprehensive Tile pelvic disruption classification [17] combines the mechanism of injury and the degree of pelvic stability and it is largely used by orthopedic surgeons in determining prognosis and treatment options, but less useful in emergency. Previous works correlated Young & Burgess pelvic fracture pattern with the risk of pelvic fracture hemorrhage [16,18]. In our study, as suggested by Eastridge et al. [12], we included in UPF pelvic fractures with major ligamentous disruption, traditionally associated with the higher risk of hemorrhage. Pelvic fractures without ligament lacerations and with low risk of bleeding, have been included in SPF group. Our results confirm that the two patterns of pelvic ring fractures, as depicted by screening radiograph, are well related to the source of hemorrhage, with an accuracy of more than 80%: abdominal bleeding is most strongly associated with SPF and retroperitoneal bleeding with UPF. Moreover, in agreement with previous works [12], the presence of an UPF has been associated with a higher mortality for hemorrhage, probably due to the higher difficulty to treat the retroperitoneal bleeding.

The choice of clinical strategy is often decisive for outcome. The most crucial early decision in a bleeding patient with abdominal trauma and pelvic fracture is to found and to treat primarily the predominant source of hemorrhage. A xipho-pubic midline laparotomy produces a 15% (about 450 ml) increase in retroperitoneal volume by disrupting normal abdominal wall tension [19]. Therefore, an unuseful celiotomy in UPF causes a significant increase of blood loss. On the other hand, a delay in celiotomy due to an unnecessary and time-consuming angiography in a major intraperitoneal injury reduces possibilities of successful surgical treatment. In general, delay in the control of predominant bleeding location increases blood volume loss, transfusion requirements, risk of coagulopathy and ultimately reduces chances of survival.

Our protocol emphasizes abdominal US or DPL to determine the need of laparotomy. We followed indications suggested by Biffl et al.[6] and ATLS guidelines [1]. Laparotomy was mandatory when US showed more than 1 cm of fluid strip or expanding or fluid in two or more spaces or when DPL evacuated more than 10 ml of blood (DPL has been used in our patients only two times, in the presence of subcutaneous emphysema). This approach has been validated in prospective clinical series [3,4] and no patient of our series required emergency celiotomy for hemorrhage after a negative US. Two delayed celiotomies were necessary to treat small bowel injuries and three for the failure of a non-operative management of splenic injuries. Conversely, no patient had a non-therapeutic celiotomy after a positive US. In three patients hemorrhage was spontaneously stopped at celiotomy, probably for the severely decreased blood pressure.

A second objective of the study was to evaluate the usefulness of CESCT in the identification of the need for emergency AE vs EF in patients with potential pelvic bleeding. Hamil et al. [20] observed in a large series of 364 pelvic fractures that the need for pelvic embolization correlated with fracture patterns that indicated major ligament disruption. The accuracy of a positive or negative "contrast extravasation sign" at CESCT for identifying patients requiring embolization has been shown higher than 95% [5,10,11]. Nevertheless, the yield of pelvic fractures angiography ranges from 43% to 64% [7] and, even in the face of hemodynamic instability, 26% to 46% of abdominal and pelvic trauma patients will have no bleeding found on angiography [21,22]. In our series, in a total of 25 patients with UPF, only 9 (29.8%) had arterial bleeding amenable to embolization, while the other 16 patients had a "venous" hemorrhage from cancellous bone fragments treatable with compression devices. It means that, even in high risk fracture patterns, angiography would be a useless and time-wasting procedure in two thirds of cases. Our clinical protocol significantly differs from those previously published [6,12], in the sense that we recommend CESCT before angiography in patients with pelvic fracture and negative or slightly positive US/DPL. This sequence may be harmful in hemodynamically compromised patients, but the use of aggressive resuscitation with early administration of blood and clotting factors associated with measures of pelvic volume reduction as pelvic binding and C-Clamp [6], as demonstrated by others [21], is the mainstay to achieve a temporary stabilization and to allow the diagnostic workup. Moreover, modern CT scan machines allow a complete study of the abdominal compartment in few minutes and no patient in the present series expired during CT scan study. Finally, CESCT is of paramount importance to document severity of intraperitoneal injuries and the need for angiographic embolization or surgery [22-24]. In our series, CESCT showed 2 hollow viscus injuries requiring surgery and 16 bleeding injuries of abdominal organs requiring AE.

Ultimately, it has been indicated that abdominal and pelvic hemorrhage in coagulopathic patients after massive infusions/transfusions, resistant to conventional treatments, may be successfully controlled by "off-label" use of recombinant factor VIIa [25,26]. We used factor VIIa only after the failure of surgical procedures and of standard medical therapy of coagulopathy (fresh frozen plasma, platelets, cryoprecipitate)[27]. In the four patients treated with factor VIIa coagulopathy was immediately reversed with stop of bleeding and transfusion needs. One of four patient died, probably due to the evolution of a severe brain trauma.

The overall mortality of our study population was 26%, while the mortality after exclusion of those who arrived in extremis was only 14%. It is difficult to compare survival data with those presented in the literature, because of different selection criteria [6,7,12]. Nevertheless, survival in the present study may be considered good, particularly if we take into account the average probability of survival (0.59 ± 0.39) calculated from anatomic and physiologic scores in our patients.

In summary, this study suggest some considerations in the strategy of management of severely injured patients with abdominal and pelvic trauma and evidence of acute bleeding: *(a) *pattern of pelvic fracture, even roughly classified by a screening radiograph, is suggestive of a significant pelvic bleeding in the majority of patients who survive initial emergency room treatment; *(b) *early application of measures of temporary pelvic closure, when needed, should be considered a completion of the initial resuscitation protocol; *(c) *once excluded the need for emergency celiotomy with US or DPL, CESCT is the best diagnostic tool to choice the appropriate way, angiography or fixation, to manage bleeding pelvic injuries; *(d) *CESCT is the best diagnostic tool to indicate the need of abdominal angiographic embolization and to diagnose initially missed hollow viscus injuries; *(e) *availability of equipped CT scan and angiographic suites closed to the emergency room and of short response time interventional radiologist is a crucial point for this diagnostic and therapeutic work-up.

**Table 3:** Therapeutic interventions in the two fracture pattern groups, 16 emergency room deaths excluded

	***UPF(%)***	***SPF(%)***	***Total (%)***	***p***
n (%)	30 (42.26)	41 (57.74)	71	
*Retroperitoneal bleeding at CESCT*	25 (83.3)	8 (19.51)	33 (46.5)	<0.01
*Retroperitoneal Arterial bleeding at CESCT*	9 (29.8)	4 (9.7)	13 (18.30)	<0.01
*Retroperitoneal Venous bleeding at CESCT*	16 (53.3)	4 (9.7)	20 (28.16)	<0.01
*Pelvic Angiographic Embolization (%)*	9 (29.8)	2 (4.87)	11 (15.49)	<0.01
*Emergency External Fixation (%)*	14 (46.6)	1 (2.43)	15 (21.12)	<0.01
*Emergency Celiotomy (%)*	4 (13.3)	14 (34.14)	18 (25.35)	<0.01
*Delayed Celiotomy (%)*	2 (6.66)	3 (7.31)	5 (7.04)	n.s.
*Angiographic embolization of abdominal parenchyma(%)*	2 (6.66)	14 (34.14)	16 (22.53)	<0.01
*Deaths (%)*	8 (26.6)	2 (4.87)	10 (14)	<0.01
